# Infancy weight gain, parental socioeconomic position, and childhood overweight and obesity: a Danish register-based cohort study

**DOI:** 10.1186/s12889-019-7537-z

**Published:** 2019-09-02

**Authors:** Torill Alise Rotevatn, Charlotte Overgaard, G. J. Melendez-Torres, Rikke Nørmark Mortensen, Line Rosenkilde Ullits, Anna Marie Balling Høstgaard, Christian Torp-Pedersen, Henrik Bøggild

**Affiliations:** 1 Public Health and Epidemiology Group, Department of Health Science and Technology, Aalborg University, Niels Jernes Vej 14, 9220 Aalborg East, Denmark; 2 DECIPHer, Cardiff School of Social Sciences, Cardiff University, 1-3 Museum Place, Cardiff, CF10 3BD Wales, UK; 3 Unit of Epidemiology and Biostatistics, Aalborg University Hospital, Søndre Skovvej 15, 9000 Aalborg, Denmark

**Keywords:** Rapid infant weight gain, Childhood overweight and obesity, Prevention, Socioeconomic position

## Abstract

**Background:**

Rapid infant weight gain (RIWG) is a very strong predictor of childhood overweight and obesity (COO). Socioeconomic position (SEP) is also related to the risk of COO and parents of different SEP may differ in their reaction to accelerated infant weight gain. Together this could lead to differences in how weight gain and COO risk relate across SEP. This study aimed to analyse possible interaction of SEP and RIWG on COO risk.

**Methods:**

A register-based longitudinal cohort study followed 19,894 healthy, term infants, born in Denmark between December 2011 and May 2015. Logistic regression models were used to estimate odds ratios (OR) of COO risk at 2 years (22–26 months) of age with 95% confidence intervals (95% CI) for categories of infancy weight gain based on changes in weight-for-age z-scores between 0 and 8–10 months of age (*slow* (<− 0.67), *mean* (− 0.67–0.67), *rapid* (> 0.67–1.34) and *very rapid* (> 1.34)). Possible multiplicative and additive interaction of SEP (based on *household income* and *maternal education*) on the relationship between infancy weight gain and COO were analysed.

**Results:**

In total, 19.1 and 15.1% experienced rapid or very rapid weight gain, respectively, and 1497 (7.5%) children were classified with COO at follow-up. These prevalences were higher in those with lower levels of SEP. Adjusted OR for COO were 3.09 (95% CI [2.66–3.59]) and 7.58 (95% CI [6.51–8.83]) for rapid and very rapid weight gain, respectively, when household income was included in the model. Results were similar in the model including maternal education. No signs of interactions were detected on a multiplicative scale. Weak signs of additive interaction were present, but these values did not reach significance.

**Conclusion:**

Both rapid and very rapid weight gain were associated with substantially higher risks of COO but these associations were not modified by SEP. This indicates that promotion of healthy weight gain should take place in all population groups irrespective of their SEP.

**Electronic supplementary material:**

The online version of this article (10.1186/s12889-019-7537-z) contains supplementary material, which is available to authorized users.

## Background

Rapid infant weight gain (RIWG), generally defined as a change of more than 0.67 standard deviations in weight-for-age z-score between two time-points during infancy, is strongly and consistently related to overweight and obesity later in life [1–4]. A recent meta-analysis found that RIWG was associated with an increased risk of both childhood and adulthood overweight and obesity (pooled odds ratio, 3.66 (95% CI [2.59 to 5.17]) in comparison with those not experiencing RIWG [4]. Although the causal pathways are not clearly known, it has been proposed that infants with RIWG have a higher level of insulin resistance, central fat deposition and general fat accumulation that increase their risk of childhood overweight and obesity (COO) [4–8]. RIWG is one of the first measurable indications of a growth trajectory that could lead to later obesity [9], which makes prevention of this weight gain pattern during infancy an important target in an early-life obesity prevention strategy.

Higher prevalence of RIWG is reported in populations of low socioeconomic position (SEP) [10, 11], and the relationship between RIWG and COO also appears stronger in observational studies comprising of populations with high proportions of low SEP individuals [12]. Socioeconomic position represents access to resources like money, power, knowledge, and social support [13], that can be used to avoid exposure to, or help to buffer the negative effects of, general risk factors and stressors [14]. In a complex interplay together with individual, social and environmental factors, these macro-levelled factors are of significance for shaping healthy behaviour [15], for instance in relation to infant feeding [16].

Short duration of breastfeeding [17], formula feeding [18, 19], early weaning [17, 20], feeding on a schedule [19], feeding using large bottles [21], and feeding high protein formula [22] are factors associated with RIWG, which typically are more commonly practised in groups of low SEP [23] and could thus help to explain the suggested stronger association between RIWG and COO in low SEP populations. Another potential explanation is social differences in views on parenting. Qualitative studies on American low-income populations [24, 25] have found that families emphasize heavy infants as indicators of good infant health and successful parenting and a strong need to be reassured that infants are provided with enough nutrition. Such views and beliefs, in addition to a potentially higher level of concerns and stressors in low SEP parents, could encourage the use of weight gain-enhancing feeding strategies to achieve infant weight gain and further accelerate infant weight gain leading to a higher risk of COO. Qualitative differences in the response for children with RIWG, in addition to socioeconomic differences in parental identification of infant overweight or obesity [26, 27], in social support for breastfeeding [28], in early weaning [29], or in the usage of these strategies to promote and prolong infant sleep [25, 30], could potentially have implications for how parents notice and respond to a rapid infancy weight gain trajectory and lead to effect modification of SEP on the association by RIWG on COO.

Several other pre-and postnatal risk factors for COO are furthermore socially patterned [23], and some, e.g. maternal pre-pregnancy obesity [31, 32], smoking during pregnancy [33–35], and occurrence of gestational diabetes [36], are proposed to cause pre-programming effects that increase the risk of both postnatal accelerated weight gain and subsequent obesity. The effect on COO risk can be amplified if several biological and social risk factors cluster and interact simultaneously, which may be the case for people with low SEP [37].

Overall, this provide a basis for evaluating whether the relationship between infancy weight gain and COO risk differs across different levels of SEP in order to identify potential high-risk groups in need of more attention by early-life obesity prevention strategies. Few studies have comprehensively tested whether the relationship between RIWG and COO risk is modified by SEP. Two studies did not identify any effect modification by maternal education [38, 39], but these studies had sample sizes around 300 and their null findings may represent a type II error. The aim of this study is therefore to analyse the relationship between RIWG and COO risk across different levels of SEP in a large cohort. We hypothesised that the association between rapid infant weight gain and risk of developing COO would be stronger for infants from parents with low SEP than for infants from parents with high SEP.

## Methods

### Data sources

This register-based longitudinal cohort study links The Children’s Database with other nationwide administrative Danish registries. The administrative register The Children’s Database has collected data on children’s (0–17 years) health status since 2009. Data is compiled at regular preventive health assessments during infancy and childhood [40, 41]. From December 2011 it has been mandatory for Danish municipalities to report data collected by health visitors, including data on child height and weight [42]. Every Danish citizen is given a unique Civil Personal Register number at birth, which enables linkage of individual-levelled data across nationwide administrative registries [43, 44]. Individual-levelled data for the study population and their parents were obtained from The Medical Birth Registry [45], The Danish Civil Registration System [44], The Income Statistics Register [46], and The Population Education Register [47].

### Population

The study population consisted of children born in Denmark between December 2011 and May 2015 and registered in The Children’s Database. Eligible infants were born at term (gestational age of 37 + 0 to 41 + 6) with birthweight > 2500 g and were registered with both their 8-, 9- or 10-months weight *and* their 22-, 23-, 24-, 25- or 26-months weight and height.

### Child anthropometric measures

Health professionals measured weight and height during planned health examinations. Weight was measured using a validated scale and reported to the nearest 0.1 kg. Height was measured recumbent until two years of age and reported to the nearest 0.5 cm [40]. We calculated weight-for-age and body mass index z-scores describing infants anthropometric standard deviations in relation to the 2006 World Health Organization (WHO) Child Growth Standards using WHO Anthro 2011, version 3.2.2 [48]. Infant weight gain was defined as the change in weight-for-age z-scores between birth and 8 to 10 months. A change of 0.67 represents moving one percentile band on a growth chart [3, 4, 49], and the change in weight gain were thus categorised as: <− 0.67 standard deviations (SD) (*slow weight gain*), − 0.67 to 0.67 SD (*mean weight gain*), > 0.67 to 1.34 SD (*rapid weight gain*), and > 1.34 SD (*very rapid weight gain*). The timing of measurement was chosen as RIWG during the first, compared to the second, year of life has shown a stronger association with later risk of overweight and obesity [4] and because a preventive health assessments including infant measurement is offered around 8–10 months of age in Denmark. The measurement closest to 9 months of age were used if several measurements were available. Implausible values (<− 6 or > 5 SD) were excluded [50]. The study outcome was childhood overweight or obesity at 2 years of age, which was defined as having a body mass index z-score of more than 2 SD [51, 52]. The measurements closest to 24 months of age were applied if several measurements were available, and implausible values (<− 5 or > 5 SD) were excluded [50].

### Socioeconomic position

Maternal education and household income served as proxies for the socioeconomic position. Level of maternal education closest to the year before or after birth was identified through The Population Education Register. This information was categorised in accordance with the International Standard Classification of Education (ISCED) 2011 [53] and adjusted to a Danish education setting as done by Ullits and colleagues [54], thus consisting of the four categories: *ISCED level 0–2* (up to and including lower secondary education)*, 4* (post-secondary non-tertiary education)*, 5–6* (short-cycle tertiary education, or Bachelor’s degree or equivalent) and *7–8* (Master’s/Doctoral degree or equivalent). Household income level was identified through The Income Statistics Register and defined as mean annual household income calculated from the two years prior to birth and divided into quartiles (*low*, *low-middle*, *high-middle* and *high*).

### Covariates

A recent review on the relationship between RIWG and COO informed the selection of relevant child and maternal covariates [4]; child sex, breastfeeding duration, birthweight, mode of delivery, maternal pre-pregnancy body mass index (BMI), maternal smoking status during pregnancy, gestational age at birth, parity, and gestational diabetes status. Data on infant sex and breastfeeding duration were identified from The Children’s Database. WHO recommend six months of exclusive breastfeeding [55], but many parents introduce complementary foods from four months of age [56]. Breastfeeding duration was thus categorised into four groups: *0–2 months, 2–4 months, 4–6 months, > 6 months*, where the cut-off at two months was set to separate short- and medium-termed breastfeeding. Information on infant birthweight, mode of delivery, maternal pre-pregnancy BMI, smoking status during pregnancy, gestational age at birth, parity and occurrence of gestational diabetes were identified in The Medical Birth Registry. Birthweight and gestational age at birth were applied to identify infant size for gestational age. Thresholds reported by Kramer and colleagues [57] were applied to define three categories: *Small-* (SGA), *appropriate-* (AGA) and *large-for-gestational age* (LGA). Maternal pre-pregnancy BMI was grouped in accordance with WHO categories: < 18.5 (underweight), 18.5–24.9 (normal weight*), 25.0–29.9 (*overweight*) 30.0–34.9 (*obesity class I*) and > 35.0 (*obesity class II + III*)* [58]. Information on maternal smoking status during pregnancy was divided into three groups: *Yes, Stopped during pregnancy, No*. Gestational age at birth was included as the week of gestation, and parity was categorised as *1*, *2* or *> 2* completed pregnancies. Mode of delivery consisted of the categories *vaginal birth* and *caesarean section*. Finally, information on infant’s birth year, region of habitation at the time of the last anthropometric measurement, and the number of anthropometric measurements registered in The Children’s Database was included in all adjusted models in order to control for potential differences.

### Statistical analysis

Between-group differences in covariate distribution across exposure categories (infancy weight gain, maternal education level and household income level) were tested using Chi-Square (X^2^) tests. Missing values were imputed by chained equations using the *mice* package in R [59]. The number of imputations was set to 33 and reflected the highest percentage of missing information on a single covariate, with 20 iterations in each chain [59]. All covariates in addition to categorical information on maternal age, birth length, and maternal ethnicity were used to inform the imputation. These additional covariates were left out of the main analyses due to the risk of over-adjustment. The administrative databases lack information on educational attainment and household income for some citizens, especially for immigrants that had their education before entering Denmark. Observations with missing data on educational attainment and income (*n* = 1123) were therefore excluded prior to imputation due to a potential violation of the *missing not at random* assumption [60]. Complete case analyses were carried out as sensitivity analyses. Furthermore, the study population and the population excluded due to missing anthropometric data were compared on sex, size for gestational age at birth, maternal education, household income, weight gain category, and COO.

Unadjusted, intermediate partly adjusted, and fully adjusted logistic regression model were used to estimate odds ratios (OR) and corresponding 95% confidence intervals (CI) of COO risks. Two intermediate models comprised of either maternal education or income level, while two fully adjusted models additionally included all covariates. This approach was chosen, as we wanted to examine the potential confounding effect of SEP alone if SEP was not found to be an effect modifier in the relationship between weight gain category and COO. Multiplicative interaction between weight gain category and SEP were tested in the fully adjusted models. Models including all covariates were stratified for SEP to further study between-group differences. Further search for high-risk groups in special need for preventive attention was conducted via tests for additive interaction between weight gain category and SEP. This was evaluated through the calculation of relative excess risk due to interaction (RERI) using the method involving categorical exposures, as described by VanderWeele and Knol [61]. RERI was calculated based on 2 × 2 tables. Maternal education and household income were dichotomised in order to ease interpretations of the results. Dichotomisation involved collapsing the two lowest and two highest categories. Comparisons between the categories of mean weight gain and rapid or very rapid weight gain were carried out. This enabled calculation of RERI for four subgroups. High level of SEP and mean weight gain category were coded as reference, as these exposure groups produced the lowest outcome risk when considered jointly [62]. These analyses were conducted on complete case data, as the method described was not developed for application on imputed data. Data management and calculation of RERI was conducted using the SAS Statistical Software package for Windows, version 9.4 (SAS Institute, Cary, NC, USA), and all further statistical analyses were conducted by using the R statistical software, version 3.5.0 [63].

### Ethics

This study was approved by The Danish Data Protection Agency (ref: 2008-58-0028, internal ref.: 2017–67). In Denmark, it is not necessary to obtain ethical approval for the use of administrative register information in research, as long as the project does not involve human biological material [64].

## Results

### Study population

**Fig. 1:**
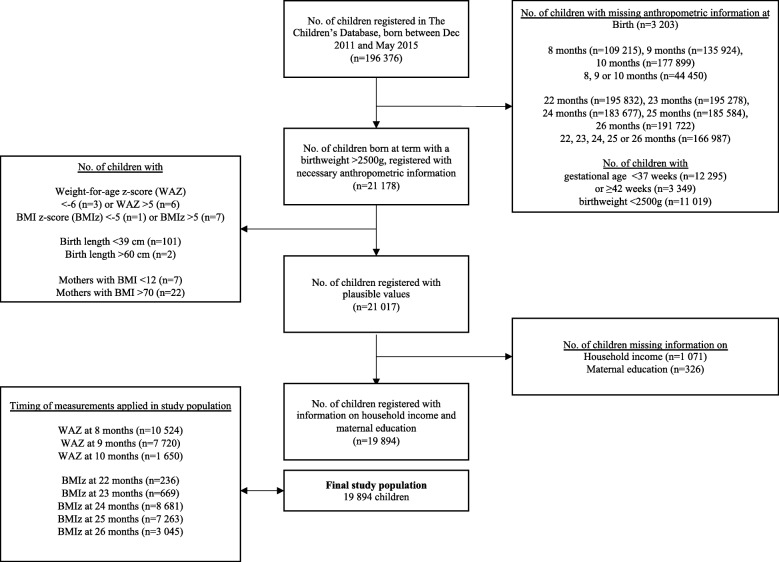
Flow chart illustrating the process of selecting the study population. Flow chart showing how the study population was arrived at and at what time the applied measurements were collected

In total, 196,376 infants born between December 2011 and May 2015 were registered at least once with their height or weight in The Children’s Database. Figure 1 illustrates how the study population of 19,894 children was arrived at. Their background information stratified by weight gain category is shown in Table 1.

**Table 1:** Table of covariate distribution on category of infancy weight gain

	Slow (*n* = 3796)	Mean (*n* = 9300)	Rapid (*n* = 3800)	Very rapid (*n* = 2998)	Total (*n* = 19,894)	X^2^ *p*-value
Child sex, n (%)						
Male	1919 (50.6)	4668 (50.2)	1930 (50.8)	1624 (54.2)	10,141 (51.0)	
Female	1877 (49.4)	4632 (49.8)	1870 (49.2)	1374 (45.8)	9753 (49.0)	0.002
Size for gestational age at birth, n (%)						
SGA	33 (0.9)	398 (4.3)	378 (10.0)	487 (16.3)	1296 (6.5)	
AGA	2574 (67.9)	7872 (84.7)	3246 (85.6)	2431 (81.2)	16,123 (81.1)	
LGA	1185 (31.2)	1022 (11.0)	170 (4.5)	74 (2.5)	2451 (12.3)	< 0.0001
Missing	4	8	6	6	24	
Gestational age at birth, n (%)						
37 weeks	52 (1.4)	296 (3.2)	262 (6.9)	392 (13.1)	1002 (5.0)	
38 weeks	284 (7.5)	1106 (11.9)	664 (17.5)	778 (26.0)	2832 (14.3)	
39 weeks	713 (18.8)	2244 (24.1)	978 (25.8)	713 (23.8)	4648 (23.4)	
40 weeks	1327 (35.0)	3057 (32.9)	1131 (29.8)	694 (23.2)	6209 (31.2)	
41 weeks	1416 (37.3)	2589 (27.9)	759 (20.0)	415 (13.9)	5179 (26.1)	< 0.0001
Missing	4	8	6	6	24	
Mode of delivery, n (%)						
Caesarean section	706 (18.6)	1751 (18.8)	788 (20.7)	719 (24.0)	3964 (19.9)	
Vaginal	3090 (81.4)	7549 (81.2)	3012 (79.3)	2279 (76.0)	15,930 (80.1)	< 0.0001
Parity, n (%)						
1	1448 (38.2)	4694 (50.6)	2191 (57.9)	1871 (62.7)	10,204 (51.4)	
2	1699 (44.8)	3410 (36.8)	1187 (31.4)	781 (26.2)	7077 (35.7)	
> 2	643 (17.0)	1171 (12.6)	408 (10.8)	334 (11.2)	2556 (12.9)	< 0.0001
Missing	6	25	14	12	57	
Maternal pre-pregnancy BMI, n (%)						
Underweight	126 (3.3)	358 (3.9)	146 (3.9)	125 (4.2)	755 (3.8)	
Normal weight	2311 (61.2)	5862 (63.4)	2363 (62.7)	1850 (62.0)	12,386 (62.7)	
Overweight	816 (21.6)	1859 (20.1)	767 (20.4)	616 (20.7)	4058 (20.5)	
Obesity I	348 (9.2)	768 (8.3)	313 (8.3)	271 (9.1)	1700 (8.6)	
Obesity II + III	173 (4.6)	396 (4.3)	179 (4.8)	120 (4.0)	868 (4.4)	0.23
Missing	22	57	32	16	127	
Maternal smoking during pregnancy, n (%)						
Yes	140 (3.7)	459 (5.0)	283 (7.5)	302 (10.2)	1184 (6.0)	
Stopped during pregnancy	108 (2.9)	318 (3.4)	141 (3.7)	116 (3.9)	683 (3.5)	
No	3516 (93.4)	8454 (91.6)	3338 (88.7)	2545 (85.9)	17,853 (90.5)	< 0.0001
Missing	32	69	38	35	174	
Gestational diabetes, n (%)						
Yes	136 (3.6)	321 (3.5)	143 (3.8)	104 (3.5)	704 (3.5)	
No	3660 (96.4)	8979 (96.5)	3657 (96.2)	2894 (96.5)	19,190 (96.5)	0.84
Duration of breastfeeding, n (%)						
0–2 months	589 (22.4)	1566 (24.9)	761 (30.2)	732 (37.6)	3648 (27.3)	
2–4 months	455 (17.3)	1121 (17.8)	461 (18.3)	348 (17.9)	2385 (17.8)	
4–6 months	1164 (44.3)	2687 (42.7)	973 (38.7)	644 (33.1)	5468 (40.9)	
> 6 months	418 (15.9)	919 (14.6)	321 (12.8)	222 (11.4)	1880 (14.0)	< 0.0001
Missing	1170	3007	1284	1052	6513	
Level of maternal education, n (%)						
ISCED 0–2	1345 (35.4)	3729 (40.1)	1744 (45.9)	1435 (47.9)	8253 (41.5)	
ISCED 4	885 (23.3)	2054 (22.1)	807 (21.2)	633 (21.1)	4379 (22.0)	
ISCED 5–6	996 (26.2)	2230 (24.0)	771 (20.3)	579 (19.3)	4576 (23.0)	
ISCED 7–8	570 (15.0)	1287 (13.8)	478 (12.6)	351 (11.7)	2686 (13.5)	< 0.0001
Household income (quartiles), n (%)						
Low	727 (19.2)	2202 (23.7)	1019 (26.8)	884 (29.5)	4832 (24.3)	
Low-middle	921 (24.3)	2331 (25.1)	965 (25.4)	741 (24.7)	4958 (24.9)	
High-middle	1052 (27.7)	2406 (25.9)	925 (24.3)	708 (23.6)	5091 (25.6)	
High	1096 (28.9)	2361 (25.4)	891 (23.4)	665 (22.2)	5013 (25.2)	< 0.0001
Overweight or obesity at follow-up, n (%)						
Yes	95 (2.5)	470 (5.1)	395 (10.4)	537 (17.9)	1497 (7.5)	
No	3701 (97.5)	8830 (94.9)	3405 (89.6)	2461 (82.1)	18,397 (92.5)	< 0.0001

Baseline characteristics of the study population (n = 19,894), stratified by infant weight gain category, shown in numbers (col%). Abbreviations: *SGA* (Small-for-gestational age), *AGA* (Appropriate-for-gestational age), *LGA* (Large-for-gestational age), *ISCED* (International Standard Classification of Education)

Of the study population, 3800 (19.1%) were classified with rapid and 2998 (15.1%) with very rapid weight gain. The distribution of all covariates except maternal pre-pregnancy BMI and occurrence of gestational diabetes differed statistical significantly across weight gain categories. Compared to infants with mean weight gain, a higher proportion of infants with rapid or very rapid weight gain were born SGA, before 39 weeks of gestation, by caesarean section, by primiparous mothers or by mothers that smoked during pregnancy, and were breastfed for less than 2 months (Table 1). The prevalence of rapid and very rapid weight gain increased with decreasing level of maternal education and household income. A social gradient was evident for rapid and very rapid weight gain when measured by either the level of maternal education or household income. Covariate distribution appeared mostly similar in both maternal education and household income (Additional files 1 and 2). The lowest prevalences of most risk factors were seen in the highest positioned groups.

### Risk of COO on infant weight gain category

A total of 1497 (7.5%) individuals were categorised with COO at follow-up at two years of age (22–26 months). The proportion increased with increasing weight gain category. 932 (15.9%) of the 5866 infants with rapid or very rapid weight gain were classified with COO. In the unadjusted model, the risk of COO was increased in infants with rapid (OR 2.18, [95%CI 1.90 to 2.51]) and very rapid weight gain (OR 4.10, [95%CI 3.59 to 4.68]) when compared to infants with mean weight gain (Table 2). These estimates remained unchanged after adjusting for SEP in the intermediate models, but they increased considerably after adjusting for covariates (Adjusted model 2a and 2b, Table 2). The risk of COO for each specific infancy weight gain category were relatively similar across levels of maternal education and household income in the stratified analyses, and confidence intervals for each specific infancy weight gain category overlapped when compared across SEP categories (Table 3). Correspondingly, no signs of interactions on a multiplicative scale were detected (maternal education: *p* = 0.89, household income: *p* = 0.24).

**Table 2:** Risk of childhood overweight and obesity calculated from logistic regression models

	Unadjusted model		Intermediate model 1a^a^		Intermediate model 1b^b^		Adjusted model 2a^c^		Adjusted model 2b^d^	
	OR	95% CI	OR	95% CI	OR	95% CI	OR	95% CI	OR	95% CI
Weight gain category										
Slow	0.48	0.39–0.60	0.49	0.39–0.61	0.49	0.39–0.61	0.27	0.21–0.34	0.27	0.21–0.34
Mean	1	–	1	–	1	–	1	–	1	–
Rapid	2.18	1.90–2.51	2.18	1.90–2.51	2.17	1.88–2.49	3.08	2.66–3.58	3.09	2.66–3.59
Very rapid	4.10	3.59–4.68	4.14	3.36–4.72	4.10	3.59–4.67	7.59	6.52–8.83	7.58	6.51–8.83
Level of maternal education										
ISCED 0–2	1.22	1.03–1.45	1.14	0.95–1.36			1.15	0.95–1.40		
ISCED 4	1.32	1.09–1.59	1.34	1.10–1.62			1.20	0.98–1.47		
ISCED 5–6	1.12	0.93–1.36	1.18	0.97–1.44			1.10	0.90–1.34		
ISCED 7–8	1	–	1	–			1	–		
Household income (quartiles)										
Low	1.40	1.21–1.63			1.28	1.10–1.50			1.34	1.13–1.59
Low-middle	1.19	1.02–1.39			1.16	0.99–1.36			1.16	0.98–1.37
High-middle	1.16	1.00–1.36			1.18	1.00–1.38			1.12	0.95–1.32
High	1	–			1	–			1	–
Child sex										
Male	1	–					1	–	1	–
Female	0.89	0.80–0.99					0.94	0.84–1.05	0.94	0.84–1.05
Size for gestational age at birth										
SGA	0.60	0.45–0.79					0.31	0.24–0.42	0.32	0.24–0.42
AGA	1	–					1	–	1	–
LGA	2.30	2.02–2.61					5.26	4.50–6.14	5.27	4.51–6.16
Parity										
1	1.01	0.90–1.13					0.88	0.77–1.00	0.84	0.74–0.96
2	1	–					1	–	1	–
> 2	1.01	0.85–1.20					0.93	0.77–1.11	0.93	0.78–1.12
Gestational age at birth										
37 weeks	1.04	0.81–1.34					0.43	0.33–0.56	0.43	0.33–0.56
38 weeks	1.07	0.91–1.27					0.62	0.52–0.74	0.62	0.52–0.74
39 weeks	0.91	0.79–1.06					0.75	0.64–0.88	0.75	0.64–0.88
40 weeks	1	–					1	–	1	–
41 weeks	1.06	0.92–1.21					1.23	1.06–1.43	1.23	1.06–1.43
Maternal pre-pregnancy BMI										
Underweight	0.52	0.35–0.78					0.56	0.37–0.84	0.55	0.37–0.83
Normal weight	1	–					1	–	1	–
Overweight	1.43	1.26–1.63					1.34	1.17–1.54	1.34	1.17–1.54
Obesity I	1.89	1.60–2.23					1.67	1.40–2.01	1.68	1.40–2.01
Obesity II + III	2.36	1.92–2.90					2.10	1.67–2.64	2.10	1.67–2.64
Gestational diabetes										
Yes	1.40	1.09–1.80					1.17	0.89–1.54	1.18	0.90–1.55
No	1	–					1	–	1	–
Maternal smoking during pregnancy										
Yes	1.48	1.22–1.80					1.22	0.98–1.51	1.16	0.93–1.43
Stopped^e^	1.54	1.20–1.97					1.45	1.11–1.89	1.40	1.08–1.83
No	1	–					1	–	1	–
Mode of delivery										
Caesarean	1.07	0.94–1.22					0.99	0.85–1.14	0.99	0.86–1.15
Vaginal	1	–					1	–	1	–
Duration of breastfeeding										
0–2 months	1.23	1.07–1.42					0.89	0.76–1.04	0.89	0.76–1.03
2–4 months	0.96	0.81–1.15					0.84	0.70–1.01	0.84	0.70–1.01
4–6 months	1	–					1	–	1	–
> 6 months	0.85	0.68–1.05					0.81	0.65–1.01	0.81	0.65–1.01

Unadjusted and adjusted odds ratios (OR) and associated 95% confidence intervals (95% CI) of overweight and obesity in the study population (n = 19,894), based on imputed data. Abbreviations: *ISCED* (International Standard Classification of Education), *SGA* (Small-for-gestational age), *AGA* (Appropriate-for-gestational age), *LGA* (Large-for-gestational age). ^a^Adjusted for maternal education and control variables (region, birth year and number of database registrations), ^b^Adjusted for household income and control variables (region, birth year and number of database registrations), ^c^Adjusted for maternal education, all covariates and control variables (region, birth year and number of database registrations), ^d^Adjusted for household income, all covariates and control variables (region, birth year and number of database registrations), ^e^Stopped during pregnancy

**Table 3:** Childhood overweight and obesity risk, stratified by level of socioeconomic position

Weight gain category	OR	95% CI	OR	95% CI	OR	95% CI	OR	95% CI
	ISCED 0–2		ISCED 4		ISCED 5–6		ISCED 7–8	
Slow	0.28	0.18–0.41	0.21	0.13–0.35	0.28	0.18–0.44	0.34	0.18–0.64
Mean	1	–	1	–	1	–	1	–
Rapid	2.99	2.38–3.77	3.02	2.23–4.09	2.88	2.09–3.96	4.14	2.61–6.56
Very rapid	7.47	5.92–9.42	7.32	5.34–10.02	6.88	4.95–9.56	11.84	7.38–19.00
	Low income quartile		Low-middle income quartile		High-middle income quartile		High income quartile	
Slow	0.26	0.15–0.43	0.16	0.09–0.27	0.36	0.24–0.54	0.29	0.18–0.47
Mean	1	–	1	–	1	–	1	–
Rapid	2.68	2.01–3.56	3.02	2.21–4.13	3.55	2.64–4.76	3.31	2.41–4.54
Very rapid	7.31	5.50–9.71	8.86	6.47–12.12	7.23	5.29–9.89	7.93	5.69–11.04

Odds ratios (OR) and 95% confidence intervals (95% CI) of overweight and obesity risk at follow-up (22–26 months) in the study population (*n* = 19,894), stratified on level of socioeconomic position, and based on fully adjusted models and imputed data. Abbreviations: *ISCED* (International Standard Classification of Education)

### Relative excess risk due to interaction for weight gain category and SEP

The values of RERI were generally positive, indicating super-additive effect modification when comparing the combined effect of low SEP and rapid or very rapid weight gain on COO risk (Table 4). Only the RERI for household income and rapid weight gain were negative (RERI: -0.19 [95%CI − 1.19 to 0.81]), but the combination of household income level and very rapid weight gain produced the largest positive RERI (RERI: 1.07 [95%CI − 0.97 to 3.11]). Although indicating super-additive effect modification in most combinations, none of these reached statistical significance.

**Table 4:** Analyses of additive interaction

Groups compared		n^a^	RERI	95% CI
*Weight gain category*	*Education level*			
Mean vs. rapid	Low vs. high	8651	0.11	− 0.82 to 1.05
Mean vs. very rapid	Low vs. high	8110	0.62	−1.32 to 2.56
*Weight gain category*	*Income level*			
Mean vs. rapid	Low vs. high	8651	−0.19	−1.19 to 0.81
Mean vs. very rapid	Low vs. high	8110	1.07	−0.97 to 3.11

Relative excess risk due to interaction (RERI) and 95% confidence intervals (95% CI) when testing additive interaction between infancy weight gain category and education level, and infancy weight gain category and income level, calculated based on the complete case population (*n* = 13,157). ^a^Number of subjects included in calculation. Calculations are based on the subjects that posit the values studied in each row

### Sensitivity analysis

Of the study population, 13,157 (66.1%) had complete information on all covariates. Frequencies of missing data were generally low except 32.7% lacking information on breastfeeding duration. Main results from the complete case analyses did not differ notably from results based on imputed data (Additional files 3 and 4). The distribution of weight gain category and COO in infants excluded due to missing anthropometric information did not differ from the distribution in the study population (Additional file 5)*.* Significant differences were observed in the distribution of infant sex, size for gestational age at birth, maternal education and household income when comparing infants with and without missing data on exposure and outcome.

## Discussion

### Key results and interpretation

Our results suggest that the relationship between rapid and very rapid infancy weight gain and COO are consistent across socioeconomic positions, implying that SEP does not modify this risk-relationship. There were weak signs of additive interaction in some combinations of SEP and rapid or very rapid weight gain on COO risk, suggesting that the public health value of preventing rapid or very rapid weight gain as a part of an early-life COO prevention strategy could be greater if carried out in groups of low compared to high SEP groups. However, these results did not reach significance. Thus, there were no support for our hypotheses proposing that the association between rapid infant weight gain and risk of developing COO were stronger in infants from parents with low than with high SEP. Thus, our comprehensive register-based cohort study confirms the results of the previous, smaller studies within this area [38, 39], and our findings suggest that activities involving prevention of RIWG and promotion of healthy infant weight gain as a part of an early-life COO prevention strategy can be relevant for all socioeconomic groups.

As in previous studies [10, 11], our results show a social gradient in infancy weight gain, where the prevalence of rapid and very rapid weight gain is higher in those with lower levels of SEP. The gradient was clearest in infants with very rapid weight gain, and higher accumulation of risk factors in infants with this weight gain pattern as well as in infants from low SEP could help to explain the social gradient. Similarly, the risk of COO increased with decreasing SEP in the unadjusted analyses. Much of this social gradient disappeared after adjusting for covariates, which indicate that a social distribution of the pre- and postnatal risk factors accounted for in this study could explain these social differences to some extent. However, risk estimates were larger for household income than for maternal eduction levels, and only the estimate for the group with the lowest income level remained significant after adjusting for covariates. This may suggest that processes linking income to health can be of special importance for early life COO risk [65]. Better access to quality food, education, health and leisure services may then be central mechanisms [65, 66].

The prevalence of COO risk factors like being born LGA, having high maternal pre-pregnancy BMI and maternal smoking during pregnancy were unevenly distributed across weight gain categories in the study population. We found that the association between infancy weight gain and COO more than doubled for both rapid and very rapid weight gain after adjusting for covariates, which suggests that other factors confounded this relationship. Our study focus was limited to considering effect modification between infancy weight gain and SEP on COO risk, and no further analyses were carried out to identify potentially responsible covariate(s). Lamb et al. [36] found that the relationship between size for gestational age and higher childhood BMI was strengthened after adjusting for infancy weight gain during the first year of life, and they explained this by suggesting that high birthweight infants still have increased risk of overweight and obesity in later childhood despite having slower weight gain trajectories during the first year of life. Notably, the risk of COO also increased considerably for LGA infants in the fully adjusted models, and this might give some support to the hypothesis proposing that the risk of COO may already commence during pregnancy through pre-programming processes that promote high birthweight [67, 68].

In the study population, a total of 15.1% experienced very rapid weight gain, and this weight gain category was associated with a seven-fold increase in COO risk when compared to infants with mean weight gain. This is consistent with the findings of a meta-analysis by Druet et al. [69], where very rapid weight gain during the first year of life, which is the equivalent to moving up two or more percentiles on a growth chart, was a particularly important predictor for later COO development. The implications of very rapid weight gain on COO risk can also be traced longer-termed, as Johnson et al. [70] observed that infants with very rapid weight gain between 0 and 3 years of age had higher mean BMI and COO risks at the ages of 11 and 14 years. This increase was higher than the increase observed in children with rapid weight gain only.

### Strengths and limitations

RIWG has commonly been studied as a dichotomization using a cut-off score of > 0.67 SD change in weight, as suggested in the seminal paper by Ong in 2000 [49]. As opposed to previous studies [38, 39], the large sample in the current study enabled a more detailed elaboration and division between rapid and very rapid weight gain in relation to population characteristics and COO risk. Our results showing significant risk differences between weight gain categories suggest that application of a simpler weight gain categorisation could result in a notable information loss. A more nuanced categorization may improve interpretation of risk estimates, as the group of reference simply consist of infants with mean weight gain rather than a combination of both slow and mean weight gain.

Another strength is having up-to-date information from national administrative databases on both infants and parents, thus enabling comprehensive confounder adjustment in multivariable regression models on contemporary data. The choice of covariates was informed by literature, but it is possible that our models only partly capture the true relationship between infancy weight gain, socioeconomic status and childhood obesity, as this relationship is fairly complex [71]. We could have conducted a more comprehensive conceptual interpretation of the relationship in e.g. a directed acyclic graph (DAG), but the traditional approach was chosen as this relationship is complicated and would possibly not be captured in a DAG.

Using data not collected for the purpose of this study can also have introduced some limitations. General practitioners are, in contrast to the health visitors, not legally obliged to register their data to the database, so local differences in data registration over time may have occurred. Information on the number of registrations for each child, birth year and administrative region were therefore included in the adjusted models in order to reduce the magnitude of this potential selection bias.

Furthermore, a high proportion of missing data was observed for the duration of breastfeeding. The task of collecting and reporting these data to The Children’s Database is generally new to health visitors, and the lack of everyday routines could have compromised data quality and completeness. However, we have no reason to believe that the data quality is compromised differently across exposure or outcome categories and possible bias would thus lead towards the null and not explain the large OR detected in our study. Furthermore, multiple imputations were made on missing values and results from complete case analyses support the robustness of our main results. We assumed that missingness of breastfeeding data were related to regional differences in registrations or birth year rather than to the length of breastfeeding itself, and multiple imputation are a more efficient method associated with less bias than complete case analyses when data are missing at random [72]. The validity of the additive interaction analyses can be questioned as these were based on complete case data, but the consistency in analyses made on both imputed and complete case data support the validity of these results.

A large proportion of infants were not registered with sufficient data in order to define exposure and outcome, why our results could be affected by selection bias. However, no significant difference was observed in the distribution of exposure or outcome when comparing populations with and without missing data, which suggest that potential effect of such bias on the results may be small. Similarly, we do not believe that differences in covariate distribution in these populations have had any significant impact on the direction of the results, as the distribution differences were small and reached significance presumably due to high power.

Finally, the generalisability of our findings is restricted to populations born at term with a birthweight of more than 2500 g. Postnatal growth of infants experiencing in-utero growth restrictions may differ from other infants due to physiological mechanisms [5], why our study population did not comprise low birthweight infants. Low birthweight is more common in groups of low SEP [23], and exclusion of low birthweight infants could thus have led to greater consistency in the relationship between birthweight, weight gain and COO across different SEP groups. It is possible that our results would appear differently if we had included low birthweight infants, and this should be assessed in future studies.

### Implications

Weak signs of additive interaction in some combinations of SEP and rapid or very rapid weight gain on COO risk were identified, but the results as a whole does show any signs of COO risk increase in any SEP groups after rapid or very rapid weight gain. Thus, there does not currently exist suffiecient evidence to suggest that promotion of healthy infant weight gain as a part of an early-life COO prevention strategy should be targeted certain socioeconomic groups, but future research should pursue the signs of additive interaction identified in our study. Anyhow, the social gradient in the prevalence of rapid and very rapid weight gain highlights that such prevention should make use of approaches that are effective in populations of low SEP. Access to resources such as money, knowledge, and power affects individuals’ ability to achieve behaviour change, and a lack of access to these resources can make individual focused behaviour change interventions less successful in populations of low SEP compared to those of high SEP [13, 73]. Conversely, interventions that integrate an intend to make changes at more upstream levels like the community, institutional, or policy level may be better strategic choices, as such interventions are more successful in reducing social inequalities in health [74, 75].

## Conclusion

Over one-third of the study population experienced rapid (> 0.67 SD) or very rapid (> 1.34 SD) weight gain within the first year of life and 7.5% were categorised with COO at 2 years of age in this Danish register-based cohort study. A social gradient was observed in both the prevalence of these weight gain patterns and the risk of COO. The risk of experiencing COO was over seven times higher for infants with very rapid compared to mean weight gain after adjusting for confounders, but there were no clear signs of modification by SEP on the relationship between rapid and very rapid weight gain category and COO risk. This suggest that prevention of RIWG and promotion of healthy infancy weight gain as a strategy for early-life obesity prevention will be valuable for all socioeconomic groups. Such prevention should make use of strategies that are effective in low SEP groups, as the prevalence of rapid and very rapid weight gain were higher in these groups.

## Additional files

Additional file 1: Tables of covariate distribution on maternal education. A table presenting how covariates are distributed across levels of maternal education. (DOCX 21 kb)

Additional file 2: Tables of covariate distribution on household income. A table presenting how covariates are distributed across levels of household income. (DOCX 21 kb)

Additional file 3: Results from logistic regression models conducted on the population with complete data. A table showing the results obtained from the logistic regression models when these are based on the population with complete data. (DOCX 22 kb)

Additional file 4: Results from logistic regression models conducted on the population with complete data, stratified by maternal education or household income. A table showing the results obtained from the logistic regression models when these are based on the population with complete data and stratified by maternal education or household income, respectively. (DOCX 14 kb)

Additional file 5: Comparison of populations with and without missing data on exposure and outcome. A table showing comparing populations with and without missing data on exposure and outcome on central variables. (DOCX 17 kb)

## Data Availability

Due to Danish law and protecting patient privacy, the combined set of data used in this study can only be made available through a trusted third party, Statistics Denmark. This state organisation holds the data used for this study. University-based Danish scientific organisations can be authorized to work with data within Statistics Denmark and such organisation can provide access to individual scientists. Requests for data may be sent to Statistics Denmark: http://www.dst.dk/en/OmDS/organisation/TelefonbogOrg.aspx?kontor=13&tlfbogsort=sektion or the Danish Data Protection Agency: https://www.datatilsynet.dk/english/the-danish-data-protection-agency/contact/.

## Abbreviations

AGA: Appropriate-for-Gestational Age
BMI: Body mass index
CI: Confidence interval
DAG: Directed acyclic graph
ISCED: International Standard Classification of Education
LGA: Large-for-Gestational Age
OR: Odds ratio
RERI: Relative excess risk due to interaction
RIWG: Rapid infant weight gain
COO: Childhood overweight and obesity
SD: Standard deviation
SEP: Socioeconomic position
SGA: Small-for-Gestational Age
WHO: World Health Organization
