# Supplementary material for: Infancy weight gain, parental socioeconomic position, and childhood overweight and obesity: a Danish register-based cohort study
Source: BMC Public Health. 2019 Sep 2;19:1209. doi: 10.1186/s12889-019-7537-z (PMC6720844; doi:10.1186/s12889-019-7537-z)
Supplement: Supplementary file 1 — Tables of covariate distribution on maternal education. A table presenting how covariates are distributed across levels of maternal education. (DOCX 21 kb) [file 12889_2019_7537_MOESM1_ESM.docx]

Additional file title: *Additional file 1: Table of covariate distribution on level of maternal education*

|  | **ISCED 0-2 (n= 8 253)** | | **ISCED 4  (n=4 379)** | **ISCED 5-6 (n= 4 576)** | **ISCED 7-8  (n= 2 686)** | **Total  (n=19 894)** | **X^2^**  **p-value** |
| --- | --- | --- | --- | --- | --- | --- | --- |
| **Child sex** |  | |  |  |  |  |  |
| Male | 4160 (50.4) | | 2215 (50.6) | 2359 (51.6) | 1407 (52.4) | 10141 (51.0) |  |
| Female | 4093 (49.6) | | 2164 (49.4) | 2217 (48.4) | 1279 (47.6) | 9753 (49.0) | 0.25 |
| **Size for gestational age at birth** | | |  |  |  |  |  |
| SGA | 665 (8.1) | | 248 (5.7) | 232 (5.1) | 151 (5.6) | 1296 (6.5) |  |
| AGA | 6769 (82.1) | | 3497 (79.9) | 3667 (80.2) | 2190 (81.7) | 16123 (81.1) |  |
| LGA | 807 (9.8) | | 630 (14.4) | 673 (14.7) | 341 (12.7) | 2451 (12.3) | <0.0001 |
| Missing | 12 | | 4 | 4 | 4 | 24 |  |
| **Gestational age at birth** | | |  |  |  |  |  |
| 37 weeks | 448 (5.4) | | 209 (4.8) | 212 (4.6) | 133 (5.0) | 1002 (5.0) |  |
| 38 weeks | 1162 (14.1) | | 694 (15.9) | 623 (13.6) | 353 (13.2) | 2832 (14.3) |  |
| 39 weeks | 1886 (22.9) | | 1033 (23.6) | 1101 (24.1) | 628 (23.4) | 4648 (23.4) |  |
| 40 weeks | 2574 (31.2) | | 1348 (30.8) | 1445 (31.6) | 842 (31.4) | 6209 (31.2) |  |
| 41 weeks | 2171 (26.3) | | 1091 (24.9) | 1191 (26.0) | 726 (27.1) | 5179 (26.1) | 0.04 |
| Missing | 12 | | 4 | 4 | 4 | 24 |  |
| **Mode of delivery** |  | |  |  |  |  |  |
| Caesarean section | 1529 (18.5) | | 962 (22.0) | 928 (20.3) | 545 (20.3) | 3964 (19.9) |  |
| Vaginal | 6724 (81.5) | | 3417 (78.0) | 3648 (79.7) | 2141 (79.7) | 15930 (80.1) | <0.0001 |
| **Parity** |  | |  |  |  |  |  |
| 1 | 6019 (73.2) | | 1721 (39.4) | 1595 (34.9) | 869 (32.4) | 10204 (51.4) |  |
| 2 | 1582 (19.2) | | 1888 (43.3) | 2221 (48.6) | 1386 (51.7) | 7077 (35.7) |  |
| >2 | 622 (7.6) | | 756 (17.3) | 752 (16.5) | 426 (15.9) | 2556 (12.9) | <0.0001 |
| Missing | 30 | | 14 | 8 | 5 | 57 |  |
| **Maternal pre-pregnancy BMI** | | |  |  |  |  |  |
| Underweight | | 354 (4.3) | 133 (3.1) | 135 (3.0) | 133 (5.0) | 755 (3.8) |  |
| Normal weight | | 5119 (62.5) | 2336 (53.8) | 2928 (64.2) | 2003 (75.1) | 12386 (62.7) |  |
| Overweight | | 1615 (19.7) | 1105 (25.4) | 956 (21.0) | 382 (14.3) | 4058 (20.5) |  |
| Obesity I | | 715 (8.7) | 499 (11.5) | 361 (7.9) | 125 (4.7) | 1700 (8.6) |  |
| Obesity II+III | | 392 (4.8) | 273 (6.3) | 179 (3.9) | 24 (0.9) | 868 (4.4) | <0.0001 |
| Missing | | 58 | 33 | 17 | 19 | 127 |  |
| **Maternal smoking during pregnancy** | | | |  |  |  |  |
| Yes | | 747 (9.1) | 320 (7.4) | 94 (2.1) | 23 (0.9) | 1184 (6.0) |  |
| Stopped during pregnancy | | 335 (4.1) | 192 (4.4) | 110 (2.4) | 46 (1.7) | 683 (3.5) |  |
| No | | 7091 (86.8) | 3824 (88.2) | 4338 (95.5) | 2600 (97.4) | 17853 (90.5) | <0.0001 |
| Missing | | 80 | 43 | 34 | 17 | 174 |  |
| **Gestational diabetes** | | |  |  |  |  |  |
| Yes | | 270 (3.3) | 191 (4.4) | 167 (3.6) | 76 (2.8) | 704 (3.5) |  |
| No | | 7983 (96.7) | 4188 (95.6) | 4409 (96.4) | 2610 (97.2) | 19190 (96.5) | 0.002 |
| **Duration of breastfeeding** | | |  |  |  |  |  |
| 0-2 months | | 1709 (31.8) | 970 (32.2) | 692 (21.9) | 277 (15.1) | 3648 (27.3) |  |
| 2-4 months | | 1019 (19.0) | 598 (19.9) | 525 (16.6) | 243 (13.2) | 2385 (17.8) |  |
| 4-6 months | | 2010 (37.4) | 1092 (36.3) | 1426 (45.2) | 940 (51.2) | 5468 (40.9) |  |
| >6 months | | 638 (11.9) | 352 (11.7) | 513 (16.3) | 377 (20.5) | 1880 (14.0) | <0.0001 |
| Missing | | 2877 | 1367 | 1420 | 849 | 6513 |  |

Additional file 1 legend: *Table showing baseline characteristics of the study population (n=19 894), stratified by level of maternal education, shown in numbers (col%). Abbreviations: ISCED (International Standard Classification of Education), SGA (Small-for-gestational age), AGA (Appropriate-for-gestational age), LGA (Large-for-gestational age).*
