# Supplementary material for: Infancy weight gain, parental socioeconomic position, and childhood overweight and obesity: a Danish register-based cohort study
Source: BMC Public Health. 2019 Sep 2;19:1209. doi: 10.1186/s12889-019-7537-z (PMC6720844; doi:10.1186/s12889-019-7537-z)
Supplement: Supplementary file 2 — Tables of covariate distribution on household income. A table presenting how covariates are distributed across levels of household income. (DOCX 21 kb) [file 12889_2019_7537_MOESM2_ESM.docx]

Additional file title: *Additional file 2: Table of covariate distribution on level of household income*

|  | **Low (n= 4 832)** | | **Low-middle  (n=4 958)** | **High-middle (n= 5 091)** | **High  (n= 5 013)** | **Total  (n=19 894)** | **X^2^**  **p-value** |
| --- | --- | --- | --- | --- | --- | --- | --- |
| **Child sex** |  | |  |  |  |  |  |
| Male | 2458 (50.9) | | 2484 (50.1) | 2643 (51.9) | 2556 (51.0) | 10141 (51.0) |  |
| Female | 2374 (49.1) | | 2474 (49.9) | 2448 (48.1) | 2457 (49.0) | 9753 (49.0) | 0.34 |
| **Size for gestational age at birth** | | |  |  |  |  |  |
| SGA | 372 (7.7) | | 342 (6.9) | 286 (5.6) | 296 (5.9) | 1296 (6.5) |  |
| AGA | 3979 (82.5) | | 4007 (80.9) | 4109 (80.8) | 4028 (80.4) | 16123 (81.1) |  |
| LGA | 473 (9.8) | | 602 (12.2) | 692 (13.6) | 684 (13.7) | 2451 (12.3) | <0.0001 |
| Missing | 8 | | 7 | 4 | 5 | 24 |  |
| **Gestational age at birth** | | |  |  |  |  |  |
| 37 weeks | 280 (5.8) | | 242 (4.9) | 227 (4.5) | 253 (5.1) | 1002 (5.0) |  |
| 38 weeks | 717 (14.9) | | 708 (14.3) | 688 (13.5) | 719 (14.4) | 2832 (14.3) |  |
| 39 weeks | 1128 (23.4) | | 1197 (24.2) | 1182 (23.2) | 1141 (22.8) | 4648 (23.4) |  |
| 40 weeks | 1475 (30.6) | | 1533 (31.0) | 1621 (31.9) | 1580 (31.5) | 6209 (31.2) |  |
| 41 weeks | 1224 (25.4) | | 1271 (25.7) | 1369 (26.9) | 1315 (26.3) | 5179 (26.1) | 0.09 |
| Missing | 8 | | 7 | 4 | 5 | 24 |  |
| **Mode of delivery** |  | |  |  |  |  |  |
| Caesarean section | 924 (19.1) | | 982 (19.8) | 1014 (19.9) | 1044 (20.8) | 3964 (19.9) |  |
| Vaginal | 3908 (80.9) | | 3976 (80.2) | 4077 (80.1) | 3969 (79.2) | 15930 (80.1) | 0.21 |
| **Parity** |  | |  |  |  |  |  |
| 1 | 3559 (73.9) | | 2897 (58.6) | 2127 (41.9) | 1621 (32.4) | 10204 (51.4) |  |
| 2 | 895 (18.6) | | 1487 (30.1) | 2247 (44.3) | 2448 (48.9) | 7077 (35.7) |  |
| >2 | 362 (7.5) | | 559 (11.3) | 699 (13.8) | 936 (18.7) | 2556 (12.9) | <0.0001 |
| Missing | 16 | | 15 | 18 | 8 | 57 |  |
| **Maternal pre-pregnancy BMI** | | |  |  |  |  |  |
| Underweight | | 241 (5.0) | 182 (3.7) | 144 (2.8) | 188 (3.8) | 755 (3.8) |  |
| Normal weight | | 2896 (60.4) | 2896 (58.8) | 3079 (60.8) | 3515 (70.6) | 12386 (62.7) |  |
| Overweight | | 958 (20.0) | 1086 (22.0) | 1146 (22.6) | 868 (17.4) | 4058 (20.5) |  |
| Obesity I | | 449 (9.4) | 494 (10.0) | 459 (9.1) | 298 (6.0) | 1700 (8.6) |  |
| Obesity II+III | | 253 (5.3) | 268 (5.4) | 236 (4.7) | 111 (2.2) | 868 (4.4) | <0.0001 |
| Missing | | 35 | 32 | 27 | 33 | 127 |  |
| **Maternal smoking during pregnancy** | | | |  |  |  |  |
| Yes | | 652 (13.6) | 301 (6.1) | 179 (3.5) | 52 (1.0) | 1184 (6.0) |  |
| Stopped during pregnancy | | 303 (6.3) | 180 (3.7) | 131 (2.6) | 69 (1.4) | 683 (3.5) |  |
| No | | 3830 (80.0) | 4428 (90.2) | 4745 (93.9) | 4850 (97.6) | 17853 (90.5) | <0.0001 |
| Missing | | 47 | 49 | 36 | 42 | 174 |  |
| **Gestational diabetes** | | |  |  |  |  |  |
| Yes | | 167 (3.5) | 197 (4.0) | 195 (3.8) | 145 (2.9) | 704 (3.5) |  |
| No | | 4665 (96.5) | 4761 (96.0) | 4896 (96.2) | 4868 (97.1) | 19190 (96.5) | 0.02 |
| **Duration of breastfeeding** | | |  |  |  |  |  |
| 0-2 months | | 1131 (35.6) | 991 (29.5) | 867 (25.2) | 659 (19.4) | 3648 (27.3) |  |
| 2-4 months | | 617 (19.4) | 603 (18.0) | 629 (18.3) | 536 (15.8) | 2385 (17.8) |  |
| 4-6 months | | 1073 (33.8) | 1295 (38.6) | 1477 (42.9) | 1623 (47.7) | 5468 (40.9) |  |
| >6 months | | 355 (11.2) | 470 (14.0) | 471 (13.7) | 584 (17.2) | 1880 (14.0) | <0.0001 |
| Missing | | 1656 | 1599 | 1647 | 1611 | 6513 |  |
|  | |  |  |  |  |  |  |

Additional file 2 legend: *Table showing baseline characteristics of the study population (n=19 894), stratified by level of household income (quartiles), shown in numbers (col%). Abbreviations: SGA (Small-for-gestational age), AGA (Appropriate-for-gestational age), LGA (Large-for-gestational age).*
