# Supplementary material for: Infancy weight gain, parental socioeconomic position, and childhood overweight and obesity: a Danish register-based cohort study
Source: BMC Public Health. 2019 Sep 2;19:1209. doi: 10.1186/s12889-019-7537-z (PMC6720844; doi:10.1186/s12889-019-7537-z)
Supplement: Supplementary file 3 — Results from logistic regression models conducted on the population with complete data. A table showing the results obtained from the logistic regression models when these are based on the population with complete data. (DOCX 22 kb) [file 12889_2019_7537_MOESM3_ESM.docx]

Additional file title: *Additional file 3: Results from logistic regression models conducted on the population with complete data.*

|  | **Unadjusted model** | | **Intermediate model 1a^a^** | | **Intermediate model 1b^b^** | | **Adjusted model 2a^c^** | | **Adjusted model 2b^d^** | |
| --- | --- | --- | --- | --- | --- | --- | --- | --- | --- | --- |
|  | **OR** | **95% CI** | **OR** | **95% CI** | **OR** | **95% CI** | **OR** | **95% CI** | **OR** | **95% CI** |
| **Weight gain category** | | | | |  |  |  |  |  |  |
| Slow | 0.49 | 0.37-0.63 | 0.49 | 0.37-0.63 | 0.49 | 0.37-0.64 | 0.27 | 0.20-0.36 | 0.27 | 0.20-0.36 |
| Mean | 1 | - | 1 | - | 1 | - | 1 | - | 1 | - |
| Rapid | 2.25 | 1.90-2.67 | 2.25 | 1.90-2.67 | 2.24 | 1.89-2.65 | 3.08 | 2.57-3.70 | 3.08 | 2.57-3.69 |
| Very rapid | 4.02 | 3.42-4.73 | 4.06 | 3.45-4.78 | 4.01 | 3.41-4.72 | 7.14 | 5.94-8.60 | 7.12 | 5.93-8.58 |
| **Level of maternal education** | | | |  |  |  |  |  |  |  |
| ISCED 0-2 | 1.34 | 1.08-1.67 | 1.28 | 1.03-1.61 |  |  | 1.27 | 1.00-1.62 |  |  |
| ISCED 4 | 1.37 | 1.09-1.74 | 1.43 | 1.12-1.83 |  |  | 1.29 | 1.00-1.66 |  |  |
| ISCED 5-6 | 1.25 | 0.99-1.58 | 1.34 | 1.05-1.71 |  |  | 1.24 | 0.97-1.59 |  |  |
| ISCED 7-8 | 1 | - | 1 | - |  |  | 1 | - |  |  |
| **Household income** (quartiles) | | |  |  |  |  |  |  |  |  |
| Low | 1.45 | 1.21-1.75 |  |  | 1.33 | 1.09-1.61 |  |  | 1.39 | 1.13-1.72 |
| Low-middle | 1.21 | 1.00-1.46 |  |  | 1.20 | 0.99-1.46 |  |  | 1.21 | 0.98-1.48 |
| High-middle | 1.20 | 1.00-1.45 |  |  | 1.22 | 1.00-1.48 |  |  | 1.16 | 0.95-1.42 |
| High | 1 | - |  |  | 1 | - |  |  | 1 | - |
| **Child sex** | | | | |  |  |  |  |  |  |
| Male | 1 | - |  |  |  |  | 1 | - | 1 | - |
| Female | 0.85 | 0.75-0.97 |  |  |  |  | 0.88 | 0.77-1.01 | 0.88 | 0.77-1.01 |
| **Size for gestational age at birth** | | | | |  |  |  |  |  |  |
| SGA | 0.66 | 0.46-0.91 |  |  |  |  | 0.35 | 0.24-0.49 | 0.35 | 0.24-0.49 |
| AGA | 1 | - |  |  |  |  | 1 | - | 1 | - |
| LGA | 1.84 | 1.84-2.53 |  |  |  |  | 4.86 | 4.01-5.88 | 4.89 | 4.03-5.92 |
| **Parity** | | | | |  |  |  |  |  |  |
| 1 | 1.06 | 0.92-1.22 |  |  |  |  | 0.92 | 0.78-1.08 | 0.88 | 0.75-1.04 |
| 2 | 1 | - |  |  |  |  | 1 | - | 1 | - |
| >2 | 1.10 | 0.89-1.34 |  |  |  |  | 1.04 | 0.83-1.29 | 1.05 | 0.84-1.30 |
| **Gestational age at birth** | | | | |  |  |  |  |  |  |
| 37 weeks | 1.02 | 0.74-1.38 |  |  |  |  | 0.44 | 0.31-0.60 | 0.43 | 0.31-0.60 |
| 38 weeks | 0.89 | 0.72-1.10 |  |  |  |  | 0.55 | 0.44-0.69 | 0.55 | 0.44-0.69 |
| 39 weeks | 0.85 | 0.71-1.02 |  |  |  |  | 0.74 | 0.61-0.90 | 0.74 | 0.61-0.89 |
| 40 weeks | 1 | - |  |  |  |  | 1 | - | 1 | - |
| 41 weeks | 1.00 | 0.85-1.18 |  |  |  |  | 1.17 | 0.98-1.39 | 1.17 | 0.98-1.40 |
| **Maternal pre-pregnancy BMI** | | | | |  |  |  |  |  |  |
| Underweight | 0.40 | 0.22-0.66 |  |  |  |  | 0.43 | 0.24-0.71 | 0.42 | 0.23-0.70 |
| Normal weight | 1 | - |  |  |  |  | 1 | - | 1 | - |
| Overweight | 1.49 | 1.27-1.74 |  |  |  |  | 1.42 | 1.20-1.68 | 1.42 | 1.20-1.68 |
| Obesity I | 1.86 | 1.51-2.28 |  |  |  |  | 1.70 | 1.36-2.12 | 1.71 | 1.36-2.12 |
| Obesity II+III | 2.10 | 1.60-2.73 |  |  |  |  | 1.81 | 1.34-2.41 | 1.81 | 1.34-2.41 |
| **Gestational diabetes** | | | | |  |  |  |  |  |  |
| Yes | 1.40 | 1.09-1.80 |  |  |  |  | 1.35 | 0.96-1.88 | 1.36 | 0.96-1.88 |
| No | 1 | - |  |  |  |  | 1 | - | 1 | - |
| **Maternal smoking during pregnancy** | | | | |  |  |  |  |  |  |
| Yes | 1.41 | 1.10-1.79 |  |  |  |  | 1.14 | 0.87-1.48 | 1.08 | 0.82-1.41 |
| Stopped^e^ | 1.64 | 1.21-2.20 |  |  |  |  | 1.47 | 1.06-2.01 | 1.42 | 1.02-1.94 |
| No | 1 | - |  |  |  |  | 1 | - | 1 | - |
| **Mode of delivery** | | | | |  |  |  |  |  |  |
| Caesarean | 0.97 | 0.82-1.14 |  |  |  |  | 0.92 | 0.76-1.10 | 0.93 | 0.77-1.11 |
| Vaginal | 1 | - |  |  |  |  | 1 | - | 1 | - |
| **Duration of breastfeeding** | | | | |  |  |  |  |  |  |
| 0-2 months | 1.19 | 1.02-1.39 |  |  |  |  | 0.88 | 0.74-1.04 | 0.87 | 0.74-1.03 |
| 2-4 months | 0.93 | 0.77-1.12 |  |  |  |  | 0.82 | 0.67-0.99 | 0.82 | 0.67-0.99 |
| 4-6 months | 1 | - |  |  |  |  | 1 | - | 1 | - |
| >6 months | 0.85 | 0.68-1.04 |  |  |  |  | 0.80 | 0.64-0.99 | 0.80 | 0.64-0.99 |

Additional file 3 legend: *Table presenting unadjusted and adjusted odds ratios (OR) and associated 95% confidence intervals (95% CI) of overweight and obesity in the complete case population (n=13 157). Abbreviations: ISCED (International Standard Classification of Education), SGA (Small-for-gestational age), AGA (Appropriate-for-gestational age), LGA (Large-for-gestational age). ^a^Adjusted for maternal education and control variables (region, birth year and number of database registrations), ^b^Adjusted for household income and control variables (region, birth year and number of database registrations), ^c^Adjusted for maternal education, all covariates and control variables (region, birth year and number of database registrations), ^d^Adjusted for household income, all covariates and control variables (region, birth year and number of database registrations), ^e^Stopped during pregnancy.*
