# Supplementary material for: Infancy weight gain, parental socioeconomic position, and childhood overweight and obesity: a Danish register-based cohort study
Source: BMC Public Health. 2019 Sep 2;19:1209. doi: 10.1186/s12889-019-7537-z (PMC6720844; doi:10.1186/s12889-019-7537-z)
Supplement: Supplementary file 4 — Results from logistic regression models conducted on the population with complete data, stratified by maternal education or household income. A table showing the results obtained from the logistic regression models when these are based on the population with complete data and stratified by maternal education or household income, respectively. (DOCX 14 kb) [file 12889_2019_7537_MOESM4_ESM.docx]

Additional file title: *Additional file 4: Childhood overweight and obesity risk, stratified by socioeconomic status (complete case analysis)*

|  | **ISCED 0-2** | | **ISCED 4** | | **ISCED 5-6** | | **ISCED 7-8** | |
| --- | --- | --- | --- | --- | --- | --- | --- | --- |
|  | OR | 95% CI | OR | 95% CI | OR | 95% CI | OR | 95% CI |
| Slow | 0.27 | 0.16-0.43 | 0.21 | 0.11-0.37 | 0.30 | 0.18-0.50 | 0.35 | 0.15-0.73 |
| Mean | 1 | - | 1 | - | 1 | - | 1 | - |
| Rapid | 3.18 | 2.40-4.21 | 2.84 | 1.94-4.13 | 2.77 | 1.88-4.06 | 4.73 | 2.71-8.37 |
| Very rapid | 6.93 | 5.22-9.24 | 7.64 | 5.23-11.22 | 6.92 | 4.67-10.28 | 9.12 | 5.02-16.83 |
|  | **Low income quartile** | | **Low-middle income quartile** | | **High-middle income quartile** | | **High income quartile** | |
|  | OR | 95% CI | OR | 95% CI | OR | 95% CI | OR | 95% CI |
| Slow | 0.23 | 0.11-0.42 | 0.14 | 0.07-0.25 | 0.40 | 0.24-0.64 | 0.34 | 0.19-0.58 |
| Mean | 1 | - | 1 | - | 1 | - | 1 | - |
| Rapid | 2.85 | 2.01-4.05 | 2.92 | 2.00-4.27 | 3.32 | 2.32-4.75 | 3.33 | 2.27-4.88 |
| Very rapid | 7.20 | 5.10-10.26 | 7.69 | 5.26-11.34 | 7.30 | 5.03-10.63 | 6.95 | 4.63-10.45 |

Additional file 4 legend: *Table presenting odds ratios (OR) and 95% confidence intervals (95% CI) from fully adjusted logistic regression models when these are stratified by the level of socioeconomic position and based on the population with complete data (n= 13 157). Abbreviations: ISCED (International Standard Classification of Education)*
