# Supplementary material for: Infancy weight gain, parental socioeconomic position, and childhood overweight and obesity: a Danish register-based cohort study
Source: BMC Public Health. 2019 Sep 2;19:1209. doi: 10.1186/s12889-019-7537-z (PMC6720844; doi:10.1186/s12889-019-7537-z)
Supplement: Supplementary file 5 — Comparison of populations with and without missing data on exposure and outcome. A table showing comparing populations with and without missing data on exposure and outcome on central variables. (DOCX 17 kb) [file 12889_2019_7537_MOESM5_ESM.docx]

Additional file title: *Additional file 5: Comparison of populations with and without missing data on exposure and outcome*

| **Variable**  (number missing) | **Lack information on WAZ between 0 and 8-10 month of age** (n (col%)) | | **X^2^**  **p-value** | **Lack information on BMIz at 22-26 month of age** (n (col%)) | | **X^2^**  **p-value** |
| --- | --- | --- | --- | --- | --- | --- |
|  | No | Yes |  | No | Yes |  |
| **Gender** (0) | | | | | | |
| Boy | 77 642 (51.1%) | 23 160 (52.1%) |  | 15 128 (51.5%) | 85 674 (51.3%) |  |
| Girl | 74 284 (48.9%) | 21 290 (47.9%) | 0.0002 | 14 261 (48.5%) | 81 313 (48.7%) | 0.59 |
| **Size for gestational age** (19 101) | | | | | | |
| SGA | 11 624 (8.5%) | 2 893 (7.2%) |  | 2 256 (8.4%) | 12 261 (8.1%) |  |
| AGA | 109 109 (79.7%) | 32 308 (79.9%) |  | 21 216 (79.4%) | 120 201 (79.8%) |  |
| LGA | 16 101 (11.8%) | 5 240 (13.0%) | <0.001 | 3 243 (12.1%) | 18 098 (12.0%) | 0.19 |
| **Level of maternal education** (15 543) | | | | | | |
| ISCED 0-2 | 57 128 (40.5%) | 14 992 (37.6%) |  | 11 642 (41.9%) | 60 478 (39.5%) |  |
| ISCED 4 | 34 762 (24.7%) | 10 368 (26.0%) |  | 6 106 (22.0%) | 39 024 (25.5%) |  |
| ISCED 5-6 | 32 729 (23.2%) | 9 865 (24.8%) |  | 6 380 (23.0%) | 36 214 (23.7%) |  |
| ISCED 7-8 | 16 384 (11.6%) | 4 605 (11.6%) | <0.001 | 3 644 (13.1%) | 17 345 (11.3%) | <0.001 |
| **Household income** (quartiles) (5 754) | | | | | | |
| Low | 41 342 (27.9%) | 12 309 (28.9%) |  | 7 216 (25.0%) | 46 435 (28.7%) |  |
| Low-middle | 38 280 (25.9%) | 10 608 (24.9%) |  | 7 353 (25.5%) | 41 535 (25.7%) |  |
| High-middle | 36 177 (24.4%) | 10 603 (24.9%) |  | 7 315 (25.4%) | 39 465 (24.4%) |  |
| High | 32 223 (21.8%) | 9 080 (21.3%) | <0.001 | 6 955 (24.1%) | 34 348 (21.2%) | <0.001 |
| **Weight gain category** (49 173) | | | | | | |
| Slow |  |  |  | 4 153 (17.6%) | 21 799 (17.3%) |  |
| Mean |  |  |  | 10 246 (43.5%) | 54 541 (43.3%) |  |
| Rapid |  |  |  | 4 338 (18.4%) | 23 275 (18.5%) |  |
| Very rapid |  |  |  | 4 807 (20.4%) | 26 484 (21.0%) | 0.17 |
| **Overweight/obesity at 2 years** (176 517) | | | | | | |
| Yes | 1 773 (7.4%) | 419 (7.5%) |  |  |  |  |
| No | 22 062 (92.6%) | 5 135 (92.5%) | 0.79 |  |  |  |

Additional file 5 legend: *Table showing distribution of infants with or without missing data needed to calculate weight-for-age (WAZ) or BMI z-score (BMIz) in the 196 376 infants registered in The Children’s Database between December 2011 and May 2015. Abbreviations: SGA (Small-for-gestational age), AGA (Appropriate-for-gestational age), LGA (Large-for-gestational age), ISCED (International Standard Classification of Education).*
